# Three-Dimensional Analysis of the Interactions between *h*LDH5 and Its Inhibitors

**DOI:** 10.3390/molecules22122217

**Published:** 2017-12-13

**Authors:** Giulio Poli, Carlotta Granchi, Mohamed Aissaoui, Filippo Minutolo, Tiziano Tuccinardi

**Affiliations:** 1 Department of Biotechnology, Chemistry and Pharmacy, University of Siena, 53100 Siena, Italy; giulio.poli@unisi.it; 2 Department of Pharmacy, University of Pisa, 56126 Pisa, Italy; carlotta.granchi@unipi.it (C.G.); filippo.minutolo@unipi.it (F.M.); 3 Department of Chemistry, University of Badji Mokhtar, Sidi Amar-Annaba-B.P. 12, Annaba 23000, Algeria; aissaouimohamed.chem@gmail.com

**Keywords:** LDH5, molecular modeling, ligand–protein interactions, LDH5 inhibitors

## Abstract

Inhibitors of human lactate dehydrogenase (*h*LDH5)—the enzyme responsible for the conversion of pyruvate to lactate coupled with oxidation of NADH to NAD^+^—are promising therapeutic agents against cancer because this enzyme is generally found to be overexpressed in most invasive cancer cells and is linked to their vitality especially under hypoxic conditions. Consequently, significant efforts have been made for the identification of small-molecule *h*LDH5 inhibitors displaying high inhibitory potencies. X-ray structure of *h*LDH5 complexes as well as molecular modeling studies contribute to identify and explain the main binding modes of *h*LDH5 inhibitors reported in literature. The purpose of this review is to analyze the main three-dimensional interactions between some of the most potent inhibitors and *h*LDH5, in order to provide useful suggestions for the design of new derivatives.

## 1. Introduction

Many malignant tumors are characterized by low oxygen conditions, which trigger the hypoxia-inducible factor 1α (HIF-1α)-mediated overexpression of a series of proteins that activate signaling pathways necessary for the survival of cancer cells under these conditions [1]. Among these proteins, the human isoform 5 of lactate dehydrogenase (*h*LDH5)—a homotetramer composed of four A subunits—plays an essential role in this process, since it catalyzes the NAD^+^-coupled conversion of pyruvate—the end product of the glycolytic pathway—to lactate, which finally is excreted out of the cells. This step allows the regeneration of the reduced cofactor NADH, thus permitting the continuation of glycolysis in cells that cannot rely on oxidative phosphorylation for energy production (“Warburg effect” or “aerobic glycolysis”). This reprogrammed metabolism leads proliferating cancer cells to produce ATP only by glycolysis, resulting in an increased glucose uptake and an elevated production of lactate, in order to adequately supply the energetic demand and the production of precursors for anabolic pathways. The acidification of the extracellular space contributes to the spread of the tumor, facilitating invasion and metastasis. *h*LDH5 was found to be overexpressed in several human tumors and it is correlated with tumor size and poor prognosis. Silencing by *sh*RNA or chemical inhibition of *h*LDH5 induces a decrease of cell proliferation and migration [2,3,4,5,6]. In the last decade, *h*LDH5 has gained a considerable attention as an attractive and potentially safe therapeutic target, and many inhibitors either synthetic [7,8,9] or isolated from natural sources [10,11] have been reported in literature. Nevertheless, inhibition of this enzyme remains a challenging goal, since the active site of *h*LDH5 comprises both a substrate binding pocket, which usually hosts the small polar structure of the substrate pyruvate, and a cofactor binding site, where NADH, which is more extended than the substrate and is composed of lipophilic as well as polar portions, is located.

## 2. X-Ray Structure Analyses

### 2.1. First Apo, Cofactor-Bound and Ternary Structures with a Substrate-Like Inhibitor

The first X-ray structures of *h*LDH5 were reported in 2001 by Read and co-workers, which determined the crystal structure of both M and H isoforms of *h*LDH in a ternary complex with the cofactor NADH and the substrate-like inhibitor oxamate (**1**) [12]. Each of the four LDH5 monomers included in the M_4_-*h*LDH structure is composed by 331 amino acids and shows an overall globular shape in which two different domains can be identified, together with the N-terminal tail that presents an extended conformation to provide a linkage for the adjacent subunit within the tetramer. The two domains form a sort of bilobed structure where a central groove, primarily hollowed within the large lobe, defines the enzyme active site (Figure S1). The larger domain is characterized by an alternation of β-strands and α-helices typical of the Rossmann-type fold, which is one of the most common super-secondary structures identified in proteins interacting with dinucleotides such as FADH, NADH, and NADPH [13]. In fact, the large domain of *h*LDH5 is responsible for the interactions with the cofactor NADH, forming its binding site, and it has been often referred to as the “co-substrate binding” domain. The smaller domain shows a different mix of α-helices and β-strands and it is responsible of key interactions with the substrate, whose binding pocket is placed at the interface between the two domains, adjacent to the nicotinamide moiety of the cofactor. While the smaller domain was found to be substantially rigid, the bigger lobe showed a very flexible segment including residues 95–110, which adopted considerably different conformations among the various *h*LDH5 monomers of the X-ray asymmetric unit (see Figure S1). In fact, this particular portion of the protein constitutes the so-called “active-site loop”, which is implied in the catalytic process and can adopt either an open or a closed conformation, thus regulating the accessibility of the enzyme active site to substrate and cofactor. The open conformation is required to allow the disposition of NADH within the binding groove, which is followed by the recruitment of the substrate as a second step of a well-ordered catalytic pathway [14]. Subsequently, the active-site loop moves towards the first α-helix of the small domain assuming the closed conformation. The closure of the active-site loop, which is the rate limiting step in the reduction of pyruvate, allows the lock of the enzyme active site and the complete assembly of the catalytic machinery, as the catalytic residue R106 approaches the substrate.

**Figure 1:**
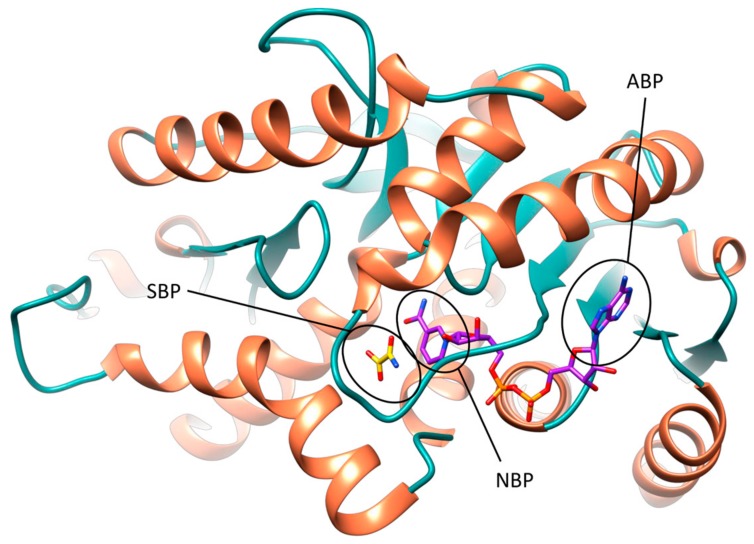
Different subpockets identified within *h*LDH5 binding groove (closed conformation). The substrate-binding pocket (SPB), nicotinamide-binding pocket (NPB) and adenine-binding pocket (ABP) are highlighted. NADH and **1** are shown in purple and yellow, respectively.

The active site of *h*LDH5 can be divided in different sub-pockets, highlighted in Figure 1. The small substrate binding pocket (SBP) and the adjacent nicotinamide binding pocket (NBP), hosting the nicotinamide moiety of NADH, are localized at the edge of the two domains and constitute the catalytic portion of the enzyme. At the other side of the binding groove, the adenine binding pocket (ABP) interacts with the cofactor adenine moiety and the linked ribose ring, while the central portion of the NADH binding site welcomes the pyrophosphate group and the other ribose unit.

In the crystal structure of *h*LDH5 reported by Read and co-workers (PDB code 1I10), the SPB is occupied by oxamate **1** (Figure S2), one of the first discovered and most known *h*LDH5 inhibitors. In chain A, where the active-site loop adopts a closed conformation, compound **1** interacts with all catalytic residues of the enzyme. In particular, the carboxylic group of the substrate-like inhibitor forms a strong salt bridge interaction with the catalytic R169, a key residue for the binding of pyruvate, and shows additional H-bonds with the hydroxyl group of T248 and the guanidine moiety of the catalytic R106 (see Figure S2A). Moreover, the ammidic oxygen of the ligand constitutes the center of an H-bond network involving the catalytic residues R106 and H193, as well as the lateral chain of N138. This latter residue extends the H-bond network to the cofactor by interacting with both hydroxyl groups of the adjacent NADH-ribose unit through its lateral and backbone amide nitrogens, whereas the positively charged H193 forms a further H-bond with the fourth catalytic residue D166, which stabilizes its charged form. Next to compound **1**, the glutamine residue Q100 belonging to the active site loop contributes to close the SBP and to shelter the inhibitor from the solvent. The inner edge of the SBP is primarily delimited by the nicotinamide moiety of the cofactor, which is sandwiched between (a) the inhibitor and the catalytic residues N138, H139, R169 (laying approximately on the same plane) from one side and (b) the lipophilic lateral chains of V31, V136, I252 from the other side. These residues delimit the NBP, which thus constitutes a rather flat cavity that can perfectly welcome planar aromatic molecular fragments such as NADH nicotinamide. This fragment of the cofactor forms hydrophobic interactions with the above mentioned residues and an H-bond with V136 backbone oxygen through its amide group, as well as a π-stacking with **1**. In the central portion of *h*LDH5 binding groove, various interactions between the cofactor and the active site loop residues can be observed. The ribose unit of NADH forms an H-bond with the backbone oxygen of A98, while the pyrophosphate group of the cofactor shows a strong ionic interaction with the guanidine moiety of R99. Moreover, two further H-bonds with A30 and V31 connect the pyrophosphate group with an α-helix of the Rossmann fold. Finally, the cofactor adenine fragment is placed within the ABP, which is delimited by V51, V53, A96, I116, I120; besides forming hydrophobic interactions with these residues, a water-bridged H-bond with the hydroxyl group of Y83 is also observed, while the connected ribose unit forms two hydrogen bonds with the carboxyl group of D52 and another one with the backbone nitrogen of G97 (see Figure S2B).

When the active-site loop adopts an open conformation as shown in chain D of the X-ray structure 1I10, the *h*LDH5 binding groove presents a quite different scenario, especially at the level of the SBP that becomes accessible to the solvent. In fact, the catalytic residue R106 shifts of more than 8 Å toward the solvent, thus losing contacts with compound **1**. Similarly, Q100 moves toward the adjacent α-helix and loses all hydrogen bonds and water-bridged interactions with both inhibitor and cofactor. However, all other H-bonds between compound **1** and catalytic residues are maintained. The H-bond with A98 is lost while the ionic interaction between the cofactor pyrophosphate group and R99 is maintained, although the residue lateral chain is shifted toward the ABP of about 4 Å. The conformation of the ABP is only marginally influenced by the movement of the active-site loop, therefore NADH shows the same interactions with the protein residues constituting this subpocket in the open and closed states of the active site. The fact that the two different conformations of the active-site loop have been identified in the different monomers of the *h*LDH5 ternary complex demonstrates that even the contemporary presence of cofactor and inhibitor is not a sufficient condition to determine the loop closure.

The first X-ray structure of *h*LDH5 in apo form was reported in 2014 by Dempster and co-workers, who also performed soaking experiments with NADH thus determining the first *h*LDH5 binary complex with the cofactor in absence of any inhibitor [15]. In both the apo (PDB code 4L4R) and the NADH-bound (PDB code 4L4S) crystal structures, the active-site loop adopts an open conformation as shown in chain D of the ternary complex in presence of **1**. The conformation of the protein in the three different X-ray structures was found to be very similar and only few differences in the orientation of the binding groove residues can be observed. As expected, the main differences can be found at the level of the active-site loop. In particular, in the apo structure the guanidine group of R99 is shifted of about four Ångstrom toward the solvent with respect to the ternary complex, because of the absence of the ionic interaction with NADH, while Q100 moves slightly toward the adjacent α-helix. Finally, a big rotational change is observed for R106, which in the apo structure is oriented toward the smaller domain and not toward the solvent as in the ternary complex. The binding of NADH through crystal soaking leads to the displacement of several water molecules occupying the binding groove of the apo structure. Nevertheless, minimal changes in both the conformation of the active-site loop and the orientation of the residues surrounding the cofactor can be observed, with the exception of R99 that moves toward NADH pyrophosphate to form the ionic interaction, as shown in the ternary complex.

### 2.2. X-Ray Structures of hLDH5 in Complex with Full Groove Binder Inhibitors

**Figure 2:**
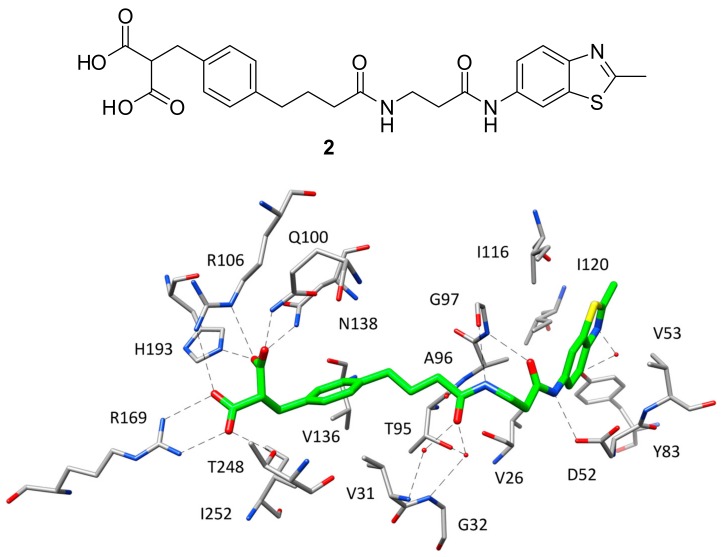
Chemical structure of **2** and X-ray structure of *h*LDH5 in complex with **2** (PDB code 4AJP). The active site residues interacting with the inhibitor are shown and the ligand–protein H-bonds are highlighted.

After the X-ray structure of *h*LDH5 in ternary complex with NADH and **1** was determined, various small molecule inhibitors have been co-crystallized with the enzyme and can be divided into different classes based on which portion of the active site they occupy within the binding complex. In fact, while most of the ligands co-crystallized with *h*LDH5 are localized at the level of the SBP, some inhibitors interact with different portions of the enzyme binding groove. Indeed, the first ligand characterized by nanomolar inhibitory activity against *h*LDH5 co-crystallized with the enzyme was specifically designed by AstraZeneca researchers to interact with all the subpockets of the active site, thus occupying the whole binding groove [16]. This ligand (compound **2**, Figure 2) was developed through a fragment-based lead generation approach started with the identification of small molecular fragments binding either the SBP/NBP or ABP of the enzyme with micromolar affinity. The most active fragment hits identified through NMR and surface plasmon resonance (SPR) screening, were also co-crystallized with rat LDH and used as a starting point for the structure-based design of a potent inhibitor through fragment-linking. The most promising ligand, showing an IC_50_ of 0.5 μM in *h*LDH5 inhibition assays, was then co-crystallized with the human enzyme (PDB code 4AJP), which adopted a closed conformation with high similarity to that observed in the ternary complex with NADH and **1**. Actually, the side chains of most of the amino acids interacting with **2** present an orientation comparable to that assumed in the ternary complex, included the residues belonging to the active-site loop. In fact, the ligand is able to efficiently mimic both the substrate structure and the different fragments of the cofactor. The SBP and NBP of the enzyme are occupied by the benzylmalonate portion of the ligand (Figure 2) that, through its two carboxylic groups, is able to establish the same H-bond network with R106, N138, R169, H193 and T248 shown by **1** in the ternary complex. Additionally, the inhibitor forms a further H-bond with Q100, while the phenyl ring mimicking the cofactor nicotinamide fragment shows hydrophobic interactions with the lateral chains of V31, V136 and I252. The central amide group of the inhibitor, placed in the portion of the binding site welcoming the cofactor pyrophosphate, is anchored to the large domain of the enzyme through a direct H-bond with the backbone oxygen of G97 and water-bridged H-bonds with the backbone nitrogens of V31 and G32, as well as with the lateral chain of T95. Finally, the methylbenzothiazole fragment of the ligand is located within the ABP and perfectly replaces the adenine moiety of NADH, showing analogue lipophilic interactions with V26, V53, A96, I116, I120 and the water-bridged H-bond with Y83. Moreover, the amide group connected to the benzothiazole ring forms additional H-bonds with the backbone of G97 and D52 carboxylic chain, thus partially mimicking the interactions shown by the cofactor ribose unit. Despite the very promising inhibitory activity of **2** in enzymatic assays, the ligand did not show any significant inhibition of lactate production in cellular assays, probably because of the diacid moiety impeding membrane permeability.

Through a fragment-based drug design approach analogue to that employed for the identification of **2**, Kohlmann and co-workers from ARIAD Pharmaceuticals developed another nanomolar inhibitor of *h*LDH5 (compound **3** of Figure S3, IC_50_ = 300 nM) that binds to the enzyme fully occupying the protein binding groove [17], as demonstrated by the corresponding ligand–protein X-ray structure (PDB code 4I9H). The biaryl moiety of the ligand is placed within the NPB and extends toward the region of the active site normally occupied by the ribose unit of the cofactor connected to its nicotinamide group (Figure S3), thus showing several hydrophobic interactions with the NBP residues. Moreover, the chlorine atom of the ligand perfectly fits a small pocket constituted by T95, V136 and S137. Despite the strong salt bridge interaction with R169, compound **3** does not form any H-bond with other catalytic residues of the enzyme. This is due to the lack of additional H-bond acceptors in the ligand, necessary to interact with H193 and N138, as well as to the fact that the active-site loop (which is not solved in all the *h*LDH5 monomers) adopts an open conformation, thus projecting R106 toward the solvent. However, a direct H-bond between the ligand and T248 hydroxyl group and a water-bridged interaction with backbone of the same residue are observed. The polydroxylated chain of the inhibitor, which showed to be important for a strong *h*LDH5 affinity, forms a network of both direct and water-mediated H-bonds with several different residues, including A30, G32, D52 and T95, while the N-arylamide group forms hydrophobic interactions with the ABP residues and the water-bridged H-bond with Y83. Moreover, the terminal carboxyarylsulfide fragment of the inhibitor binds a previously unexplored portion of the enzyme larger domain, forming additional lipophilic interactions with the ABP residues and F119, as well as a salt bridge with R112. This latter interaction, which virtually replaces the missing H-bonds with the enzyme catalytic residues, provides a fundamental contribute to the enzymatic affinity, as demonstrated by the SAR data. In contrast to **2**, compound **3** proved to reduce extracellular lactate levels by 56–68% (after one to four hours) in a Burkitt’s lymphoma cell line, at a concentration of 200 μM, thus suggesting that the disposition of the two acidic functionalities at the edges of the ligand allowed a better cell membrane permeability.

### 2.3. X-Ray Structures of hLDH5 in Complex with Inhibitors Binding the SBP

**Figure 3:**
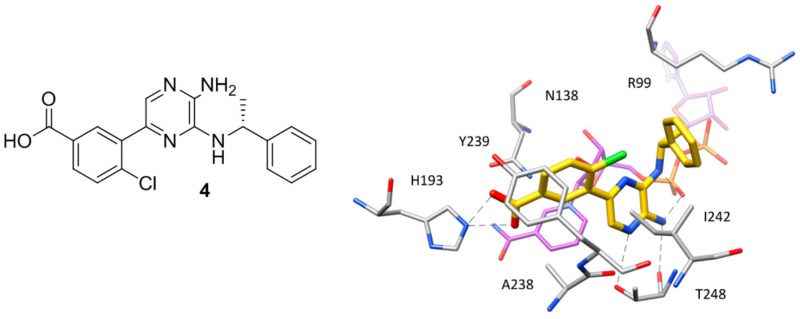
Chemical structure of **4** and X-ray structure of *h*LDH5 in complex with compound **4** (PDB code 4M49). The cofactor and active site residues interacting with the inhibitor are shown and the ligand–protein H-bonds are highlighted.

**Figure 4:**
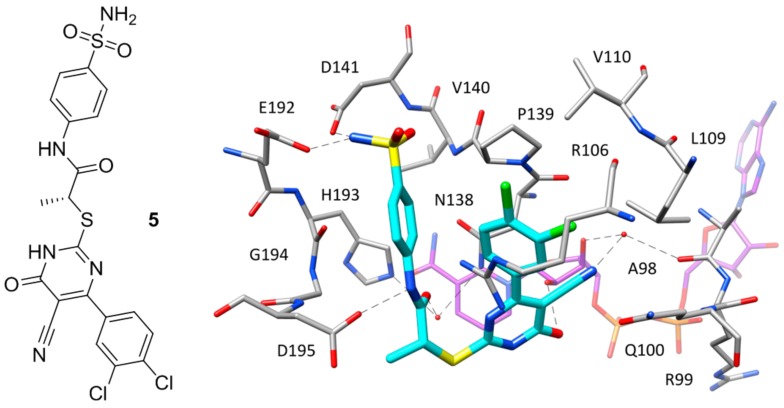
Chemical structure of **5** and X-ray structure of *h*LDH5 in complex with compound **5** (PDB code 4JNK). The cofactor and active site residues interacting with the inhibitor are shown and the ligand–protein H-bonds are highlighted.

Except for the two inhibitors discussed above, no other compound co-crystallized with *h*LDH5 occupies the whole binding groove of the enzyme. The majority of these ligands bind the enzyme at the level of the SBP without competing with NADH and have been crystallized within ternary complexes in the presence of the cofactor, with the enzyme showing an open conformation of the active-site loop. Such compounds mainly constitute the fruits of high-throughput screening campaigns performed at Genetech on the Genetech/Roche corporate compound collection through biochemical, SPR and saturation transfer difference NMR (STD-NMR) experiments, followed by hit-to-lead optimization studies. One of the first ligands belonging to this group is a low micromolar inhibitor of *h*LDH5 (IC_50_ = 2 μM) characterized by a 2-amino-5-aryl-pyrazine scaffold (compound **4**, Figure 3), which has been co-crystallized with *h*LDH5 in a ternary complex with NADH in 2013 [18]. The ligand binds the enzyme in an open conformation and occupies the SBP forming an ionic interaction with the positively charged H193 through its carboxylic group (Figure 3). The salt bridge with the catalytic residue H193 was found to be a key ligand–protein interaction of this inhibitor. In fact, derivatives in which the carboxylic group was replaced by other functionalities were found to be inactive and a drop of potency was observed for analogues where the carboxylic group could not assume the optimal orientation to interact with H193. Additional interactions fundamental for the activity of the ligand are formed by its 2-aminopyrazine scaffold, which is anchored to both the protein and the cofactor through a series of H-bonds. In particular, two H-bonds are established with backbone and side chain of T248, while two further H-bonds involve the pyrophosphate group of NADH. Finally, the ligand takes hydrophobic contacts with the nicotinamide-ribose portion of the cofactor and the residues delimiting the solvent exposed side of the SBP such as A238 and I242, as well as with Y239 and the side chain of R99. Although compound **4** was not tested for cellular activity, an analogue compound with better inhibitory potency showed only minimal cellular activity probably due to the very high plasma protein binding. Another ligand whose X-ray structure in complex with *h*LDH5 was reported in 2013 by the same research group derives from a low micromolar inhibitor with a 2-thio-6-oxo-1,6-dihydropyrimidine central core identified by high throughput screening [19]. After submicromolar derivatives of the initial hit were identified, one of the most potent compounds (compound **5** of Figure 4, IC_50_ = 0.75 μM) was co-crystallized with *h*LDH5 in open conformation and NADH (PDB code 4JNK). The inhibitor represents a rather atypical ligand that binds the enzyme in a U-shaped bioactive conformation and, despite the strong potency, does not show any direct H-bond interaction with the enzyme catalytic residues (Figure 4).

Nevertheless, the compound shows a wide network of water-bridged H-bonds, one of which connects the amide carbonyl of the ligand with N138 and H193, while a direct H-bond between D195 and the ligand amide nitrogen is observed. The central core of the compound forms an H-bond with a hydroxyl group of the close ribose unit of NADH, whereas a second water-bridged H-bond network is found between the same moiety of the cofactor, the carbonyl oxygen of A98, and the ligand nitrile group, which showed to be fundamental to maintaining a strong inhibitory activity, maybe also due to its possible influence in the tautomer distribution or in the p*K*_a_ of the compound. Moreover, this particular inhibitor occupies additional regions of the binding groove located above the SBP, thus forming unique ligand–protein interactions. In particular, the *o*-dichlorophenyl ring of the compound well fits the hydrophobic pocket mainly delimited by L109, V110 and P139. Finally, the phenylsulphonamide portion of the compound is placed at the edge of the two protein domains and forms H-bond interactions with D141 and E192. Despite the promising activity of compound **5**, neither this ligand nor the other two analogues with nanomolar potency tested for cellular activity inhibited the production of lactate in HCC1954 cells in concentrations up to 50 μM.

**Figure 5:**
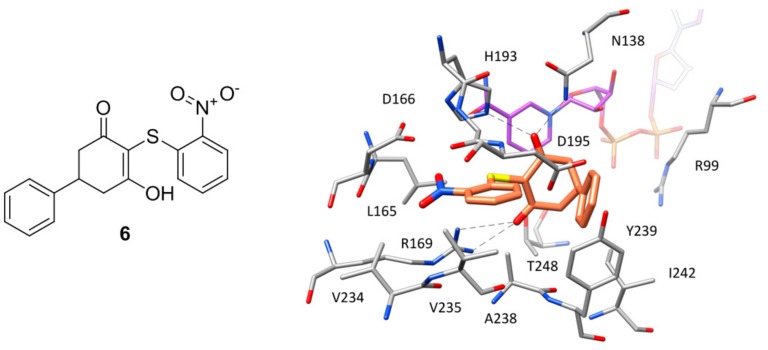
Chemical structure of **6** and X-ray structure of *h*LDH5 in complex with compound **6** (PDB code 4QO7). The cofactor and active site residues interacting with the inhibitor are shown and the ligand–protein H-bonds are highlighted.

A series of *h*LDH5 inhibitors of which several different representatives have been co-crystallized with the enzyme, was developed starting from a further high-throughput screening hit reported by Dragovich and co-workers [20]. The starting hit (compound **6** of Figure 5) is a small molecule characterized by the presence of a 3-hydroxy-2-mercaptocyclohex-2-enone scaffold, which was found to inhibit *h*LDH5 with low micromolar potency (biochemical IC_50_ = 1.7 μM, SPR-derived *K*_d_ = 3.5 μM). More than 50 analogues of the compound were synthesized with the aim of obtaining SAR data and identifying more potent ligands; nevertheless, most of derivatives were found to be inactive and very few of them maintained a good inhibitory activity. A crystal structure of compound **6** in complex with *h*LDH5 was thus obtained (PDB code 4QO7) in order to facilitate the design of analogues with improved potency. This X-ray structure showed that the ligand bound the SBP of the enzyme in open conformation and in presence of NADH, thus confirming the results of SPR experiments displaying a more than 200-fold reduced activity in absence of cofactor. In fact, the hydroxy-cycloexenone scaffold of compound **6** forms hydrophobic interactions with the nicotinamide ring of NADH (Figure 5), being located exactly in the position of the substrate (or substrate-like inhibitors). Actually, due to its low p*K*_a_ (2.63), the ligand assumes a negatively charged enolate form at physiological conditions; therefore, its central core not only sterically replaces the substrate, but also acts as a carboxylic acid mimic, forming ionic and H-bond interactions with the catalytic residues N138, R169 and H193. The 2-nitrophenyl group of the inhibitor well fits a rather flat and lipophilic pocket delimited by L165, D166, R169, D195, V234, V235 and A238, which would not be able to welcome substituents of the phenyl ring much bulkier than the nitro group or placed on different positions. This observation explained why only the 2-chloro and 2-cyano analogues of compound **6** maintained a low micromolar activity. Finally, the phenyl ring of the ligand located axially with respect to its core forms edge-to-face interactions with both the 2-nitrophenyl ring and phenol moiety of Y239, as well as additional hydrophobic interactions with A238 and L242, which should compensate for the high-energy conformation adopted to bind the enzyme.

Based on the X-ray structure, a further series of 2-chloro and 2-cyano analogues of compound **6** (in which the nitro group was thus replaced) bearing various substituents on the axial phenyl ring were synthesized in order to explore possible modifications on this portion of the ligand. While meta- and para-substituted derivatives showed lower inhibitory activities, 2,6-disubstituted compounds were found to be generally more potent than the unsubstituted analogues, with some compounds reaching submicromolar inhibitory activities. One of these derivatives, compound **7** of Figure S4, presenting a 2,6-dichlorophenyl ring and a further chlorine atom in place of the nitro group, showed an IC_50_ value for *h*LDH5 inhibition of 0.87 μM (*K*_d_ = 1.8 μM) and was co-crystallized with the enzyme (PDB code 4QO8). This compound bound the open conformation of the protein maintaining the binding mode of the parent ligand (Figure S4), with the exception of the 2,6-dichlorophenyl moiety that was surprisingly oriented in equatorial position with respect to the cycloexenone core. In fact, by assuming this conformation, the ligand was able to form better hydrophobic interactions with the ribose-nicotinamide portion of the cofactor, with I242 and R99, as well as to avoid intramolecular steric clashes.

Although compound **7** showed good inhibitory potency, this analogue was not found to inhibit the production of lactate in HCC1954 cells at concentrations of up to 50 μM. For this reason, further derivatives were synthesized in order to improve the activity of the ligand. The first structural modifications were still focused on the 2,6-dichloriphenyl ring and were aimed at maximizing the interactions of the ligand with NADH by occupying the portion of the binding groove between the cofactor and the residues of the active-site loop [8]. In this respect, compound **8**, bearing a 4-aminotetrahydropiranyl group showed a 14.5-fold improved inhibitory activity (IC_50_ = 60 nM) and was thus co-crystallized with *h*LDH5 (Figure S5, PDB code 4R69). The X-ray structure revealed that the newly inserted substituent was unexpectedly oriented toward the solvent, instead of pointing toward and interacting with NADH. The potency gain observed for the ligand compared to compound **7** can be partially explained in terms of improved interactions with the protein residues. In fact, although the ligand binds an open conformation of the enzyme, some residues belonging to the active-site loop (which is however not completely solved) are placed closer to the inhibitor with respect to the co-crystal structures of the previous analogues. In particular, the lateral chain of Q100 wraps on the 4-aminotetrahydropiranyl group of the ligand and strengthens its interactions with Y239 and I242.

However, given the impossibility of perfectly deciphering all ligand–protein interactions responsible of the activity improvement due to the disordered active-site loop, further structural modifications of these ligands focused on the other phenyl ring of compound **7**, with the aim of filling the unoccupied space lying between the catalytic residues H193, Q138 from one side and D195, Y239 from the other. Compound **9** (Figure S6), one of the new ligands synthesized, bearing a phenetyl ester moiety, was found to be 10-fold more potent than compound **8** (IC_50_ = 6 nM), and was co-crystallized with *h*LDH5. In this case, the newly introduced substituent showed to be placed exactly in the desired spot (Figure S6) and was found to form an additional H-bond with the backbone nitrogen of D195. Moreover, although the ligand still binds the enzyme in open conformation, the side chain of R106 (usually disordered) stably interacts with D195 and Y239, thus closing the SPB, sheltering the ligand from the solvent and forming a cation–π interaction that further contributes to the activity of the inhibitor. Unfortunately, despite the strong increase of potency with respect to the initial representatives of this series of inhibitors, even the most potent compounds such as compound **9** were unable to inhibit lactate production in MIA PaCa2 cells under serum conditions (10% fetal bovine serum) at concentrations up to 10 μM, probably due to a high plasma protein binding and low cell permeability, as suggested by in vitro ADME profiling.

In parallel to the SAR studies reported above, the series of compounds was expanded by synthesizing analogues bearing modifications on the central core [9]. The results showed that a dihydropyrone scaffold could endow the ligands with slightly better apparent cell permeability and slightly lower human plasma protein binding without negatively affecting their inhibitory activity. A new series of 3,6-disubstituted dihydropyrones was thus designed based on the structure of compound **6** with the aim of targeting the portion of the enzyme binding groove between the cofactor and the residues of the active-site loop. Although no potency improvement with respect to the parent ligand was obtained, the analogues with a second substituent at C6 position of the central core maintained a low micromolar activity, suggesting that the double substitution was well tolerated. These results stimulated the synthesis of spyrocyclic dihydropyrones, among which compound **10** of Figure S7 showed nanomolar inhibitory activity against *h*LDH5 (IC_50_ = 360 nM) and was co-crystallized with the enzyme (PDB code 4RLS). The dihydropyrone core of the ligand maintained the same H-bond interactions with the catalytic residues of the enzyme already shown by the previously reported cycloexenones (Figure S7); moreover, the endocyclic oxygen of the inhibitor formed a further H-bond with the side chain of T248, which contributed to its improved activity with respect to the parent compound. The 2-chlorophenyl ring of the ligand is located in the predominantly lipophilic pocket delimited by L165, D166, R169, D195, V234, V235 and A238, assuming the disposition observed for the previous compounds. Finally, although the active-site loop of the enzyme in open conformation is not completely solved in the X-ray structure, it is possible to see that R99 interacts with the indane group of the inhibitor, which takes hydrophobic contacts with both the nicotinamide and ribose moiety of the cofactor, thus actually occupying the free space between the flexible loop and NADH. Although all these efforts, the newly reported compounds were found to be unable to inhibit the production of lactate in MCF7 cells at concentrations up to 50 μM. The lack of cellular activity was still partially due to the high rate of plasma protein binding, since the ligands caused a reduction in lactate production when tested in absence of serum. Moreover, in vitro ADME studies highlighted a low MDCK cell permeability for these compounds.

As the unfavorable pharmacokinetic properties of the compounds were attributed to the acidic nature of the core, the authors decided to test the effect of an additional modification in the ligand central scaffold by synthesizing a series of hydroxylactam inhibitors. Therefore, a new series of 6,6-disubstituted hydroxylactam derivatives was synthesized, among which various compounds bearing a thiophenyl ring at C6 showed very promising *h*LDH5 inhibitory activities (IC_50_ < 100 nM) [7]. One of the most potent inhibitors of the series, compound **11** of Figure S8 (IC_50_ = 42 nM), was co-crystallized with the enzyme (PDB code 5IXS) and showed to maintain a binding mode comparable to that observed for the cycloexenones and dihydropyrones, forming the same H-bonds with the catalytic residues N138, R169, H193 and placing the conserved 2-chlorophenyl group within the pocket delimited by L165, D166, R169, D195, V234, V235 and A238 (Figure S8). The X-ray structure demonstrated that two different aryl substituents linked to the C6 position of the ligand core could be perfectly welcomed within the SPB of the enzyme. In particular, the axial thiophenyl ring shows the same disposition observed for the phenyl ring of compound **6** thus forming the same intramolecular and ligand–protein edge-to-face interactions and taking hydrophobic contacts with A238 and I242, whereas the phenol ring is sandwiched between the side chain of I242 and the ribose ring of the cofactor, forming also additional interactions with the side chain of R99. Moreover, the phenolic hydroxyl group is involved in a water-bridged H-bond network with the backbone oxygen of T248 and NADH pyrophosphate group.

The co-crystal structure was used to guide the design of a new series of 6-thiophenyl-6-arly-hydroxylactams bearing differently substituted phenyl rings at C6. Since SAR analyses demonstrated that the H-bond network allowed by the phenol group was not providing a strong contribute to the ligand inhibitory activity, substituents were introduced in both para- and meta-position of the phenyl ring. This optimization stage allowed to identify single-digit nanomolar inhibitors, among which compound **12** of Figure S9 was co-crystallized with *h*LDH5. Being a chiral compound, the X-ray structures of both stereoisomers of the ligand in complex with the enzyme were solved, in order to provide a rational to explain the different activity of the (*R*) and (*S*) stereoisomers, for which IC_50_ values of 3 nM and 55 nM, respectively, were determined (PDB codes 4ZVV and 5IXY, respectively). The two compounds showed and almost identical disposition within the SPB of the enzyme, with the only difference constituted by the orientation of the lactam core, which is rotated 180° (Figure S9). In particular, the core of the eutomer (*R*-**12**) presents the same orientation observed in compound **11**, with the lactam nitrogen pointing in the direction of I242 side chain (Figure S9A). On the contrary, the lactam nitrogen of the anomer (*S*-**12**) points toward the amide nitrogen of N138 (Figure S9B), thus forming possible unfavorable dipole-dipole interactions that could partially justify the reduced activity of the enantiomer. Moreover, the compound shows slight steric clashes with T248, whose hydroxyl group is placed close to the endocyclic carbon of the ligand core. The three side moieties of the ligands maintain the disposition displayed for compound **11**, with the equatorial phenyl ring still sandwiched between NADH ribose unit and the side chain of I242, while the morpholine moiety is placed in a solvent exposed side pocket delimited by G246 and Y247, the pyrophosphate group of the cofactor and R99. In the two X-ray structures the active-site loop is completely solved in an open conformation where R99 is shifted of about 3.5 Å toward the ABP, compared to the co-crystal complex of compound **11**, due to the presence of the morpholine cycle that forms polar interactions with the guanidine moiety of the residue justifying the improved activity with respect to the parent compound. Compound *R*-**12**, proved to be endowed with good cellular potency, being able to reduce lactate production in MIA PaCa2 cells and in presence of 10% fetal bovine serum, with an IC_50_ of 0.67 μM. Indeed, the compound showed improved cell permeability and lower plasma protein binding with respect to the parent compounds and the other potent ligands of the series. Moreover, the compound was found to inhibit the proliferation of 37 out of 347 cancer cell lines with IC_50_ values lower than five micromolar, and demonstrated good oral bioavailability in mice, thus representing the first *h*LDH5 inhibitor with adequate cellular potency and pharmacokinetic properties to allow further in vivo studies, where the compound was used to probe the role of *h*LDH5 in tumor growth in vitro and in vivo. The ligand was able to significantly modulate the levels of glycolysis-associated metabolites in MIA PaCa2 cells after six hours of exposure and to stop cell proliferation by inhibiting the glycolysis [21].

### 2.4. X-Ray Structures of hLDH5 in Complex with Small Moleucles Binding the SBP

**Figure 6:**
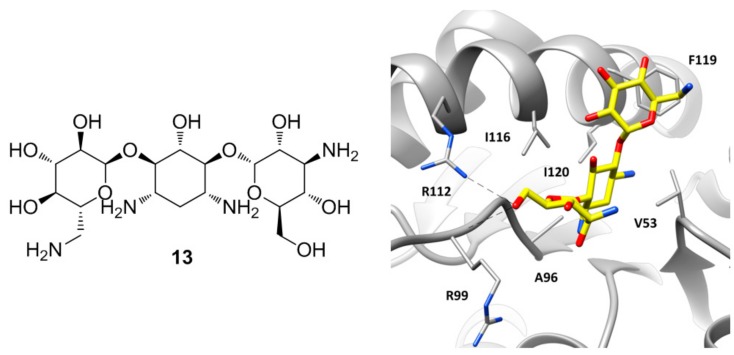
Chemical structure of kanamycin (**13**) and X-ray structure of *h*LDH5 in complex with **13**, (PDB code 4OKN). The active site residues interacting with the inhibitor are shown and the ligand–protein H-bonds are highlighted.

A particular group of *h*LDH5 X-ray structures has been reported in 2015 by Kolappan and co-workers, who aimed at solving new apo, cofactor-bound and ligand-bound co-crystal structures of *h*LDH5 [22]. The apo structure of the protein (PDB code 4OJN) was found to be overall identical to that previously reported by Dempster and collaborators (PDB code 4L4R), since the backbone atoms of the protein in chain A of both structures presented an average root-mean square deviation of 0.46 Å. However, the X-ray structure cannot be considered an actual apo structure of *h*LDH5, due to the presence of a PEG 400 polyethylene glycol molecule deriving from the crystallization mixture, which is bound at the edge of the two domains of all protein chains (within the SBP/NBP) by assuming a C-shaped conformation and forming several hydrophobic contacts with the surrounding residues, as well as some H-bonds with N138. Similar interferences from components of the crystallization mixtures occurred in the attempt to solve the binary NADH-*h*LDH5 complex, as the obtained X-ray structure showed also the presence of an oxalate molecule in seven out of the eight chains of the crystal (PDB code 4OKN). In fact, the compound is a pyruvate mimic and binds the SBP of the enzyme in presence of the cofactor by assuming the same disposition shown by the oxamate molecule in the ternary complex reported by Read and co-workers (PDB code 1I10) [12], forming also the H-bonds with R106 allowed by the closed conformation of the active-site loop. However, the peculiarity of this X-ray structure lies in chain B, where besides the presence of oxalate in the SBP of the protein, the cofactor is missing and a molecule of kanamycin is localized at the level of the ABP (Figure 6). Kanamycin (**13**, Figure 6) binds the large domain of the enzyme assuming a bent conformation, where the central ring of the molecule is placed within ABP in the position of the adenine fragment of the cofactor, thus forming hydrophobic interactions with V53, A96, I116, and I120, while the side rings are pointing toward the solvent. One of the glycan rings forms H-bond interactions with the guanidine moiety of R112 and the backbone of R99. In fact, in this chain the active site loop adopts an open conformation and both residues are shifted toward the ABP with respect to the other chains. The other sugar ring of kanamycin partially occupies the side pocket of the enzyme already explored by compound **11**, forming hydrophobic interactions with I116 and in particular with F119.

The same authors reported the X-ray structure of *h*LDH5 in complex with two well-known and structurally similar inhibitors with a quinolone central scaffold (compounds **14** and **15** of Figure S10), which showed low micromolar inhibitory activity (with IC_50_ values of 14.4 and 2.2 μM, respectively). Interestingly, the two compounds were found to be competitive with respect to NADH but not with respect to pyruvate. In agreement with the experimental data, the co-crystal structures of the ligands with *h*LDH5 (PDB codes 4QT0 and 4QSM) showed that the compounds bound the larger domain of the enzyme (in open conformation) at level of the ABP similarly to **13**, with the quinolone fragment replacing the adenine moiety of the cofactor and the lateral aryl groups pointing toward the solvent with the same disposition of the side rings of kanamycin (Figure S10). The two compounds, which only differ for the C7-substituent on the quinolone core, show an almost identical binding mode, with the central core sandwiched between V53 and I116, while the dimethoxypyridine ring forms a perfect π–π stacking with the side chain of F119 and additional lipophilic interactions with I116. The aminobenzoic moiety of both ligands forms ionic interactions with R112 and additional H-bonds with the backbone of R99. This latter residue forms a cation–π interaction with the phenyl ring of compound **14**, while in the **15**-bound structure, R99 is shifted of about six Ångstrom away toward the solvent. Finally, both the amide group of **14** and the sulfonamide group of **15** form H-bond interactions with the backbone of G97 and the carboxylic chain of D52.

## 3. Docking Analyses

Beyond the ligand-LDH5 complexes analyzed by means of X-ray crystallographic studies, during the last years a certain number of LDH inhibitors have also been analyzed by applying molecular modeling methods. The reported results do not have the same reliability of the X-ray crystallographic studies; however, they are able to give a hypothetical binding disposition for these inhibitors.

**Figure 7:**
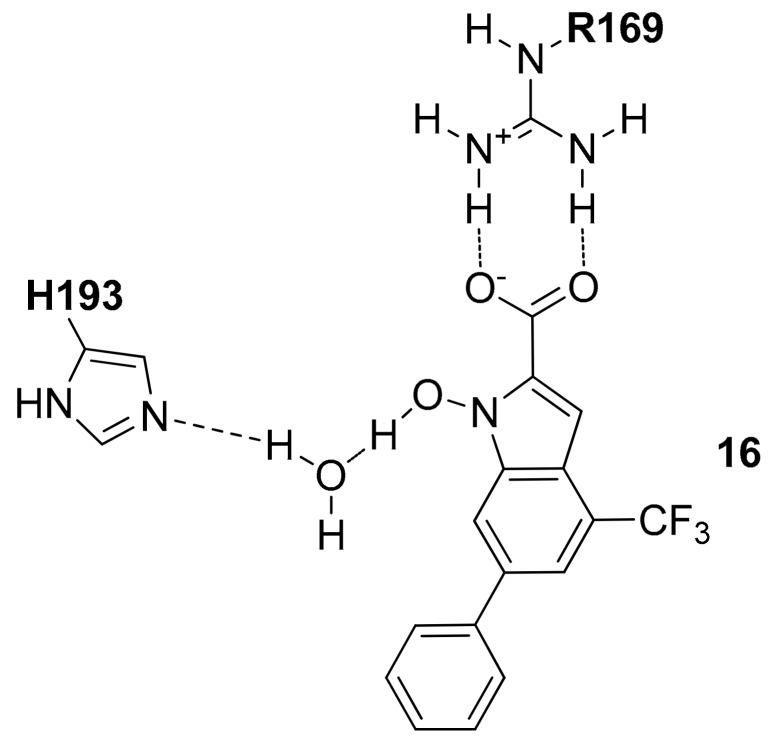
Schematic 2D representation of the **16**–*h*LDH5 H-bond interactions.

Starting from 2011, the potential use of the *N*-hydroxy-1*H*-indole-2-carboxylic acid as the main scaffold of new *h*LDH5 inhibitors was explored, by hypothesizing that this fragment is able to interact with the open form of the receptor occupying both the pyruvate and NADH binding sites [23]. The authors explored the effects of different substituents on the N-OH-indole ring with the aim of improving the inhibitory activity of the resulting compounds, introducing for example triazole moieties [24], substituted phenyl rings [25], and sulfonamide-containing substituents [26] and among all these compounds, the most active one showed a trifluoromethyl group in position 4 and a phenyl ring in position 6 (compound **16**, *h*LDH5 *K*_i_ = 8.9 µM in competition with NADH and *K*_i_ = 4.7 µM in competition with pyruvate) [27]. As shown in Figure 7, by applying a mixed docking/molecular dynamics (MD) calculation they hypothesized that the carboxylic group forms a strong interaction with R169 and T248, whereas the N-hydroxy group shows an H-bond interaction with the nitrogen backbone of T248 and a water molecule that mediates the interaction with the catalytic H193.

Starting from this compound, the authors also tried to glycosylate the N-hydroxyl group adding a glucose moiety in order to improve the uptake in cancer cells. As expected, the resulting compound showed a general loss of inhibition activity, compared to that of compound **16**, because of the loss of the interactions of the hydroxyl group. However, due to formations of H-bonds between the glucose, the side chain of N138 and the backbone of V136 and S137, the resulting compound showed only about a two-fold decrease of inhibitory activity [28].

**Figure 8:**
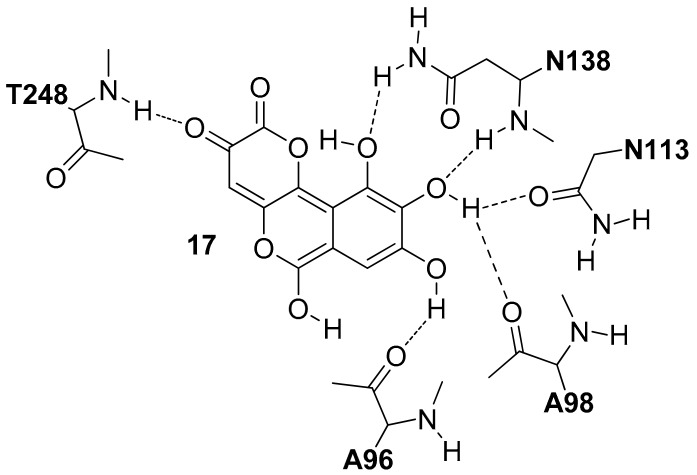
Schematic 2D representation of the **17**–*h*LDH5 H-bond interactions.

In 2012, Di Stefano and co-workers identified Galloflavin (**17**) as a new LDH5 inhibitor by applying a structure-based virtual screening (VS) protocol on the NCI Diversity Set [29]. The authors suggested that this molecule interacted in the open form of the enzyme occupying both the pyruvate and NADH binding sites (**17**, *h*LDH5 *K*_i_ = 56.0 µM in competition with NADH and *K*_i_ = 5.46 µM in competition with pyruvate). As shown in Figure 8, this compound forms a large number of hydrogen bonds. One of the two carbonyl oxygens of **17** forms an H-bond with the T248 backbone, whereas three hydroxyl groups establish H-bond interactions with the backbone and sidechain of N138, the side chain of N113 and the backbone of A96 and A98.

In 2015 Tuccinardi and co-workers developed a fully automated docking-based virtual screening platform considering different protein conformations and the consensus docking strategy [30]. By applying this approach, they screened the University of Illinois Marvel library, a collection of about 10,000 compounds and identified two compounds able to interact with the enzyme [31]. Compound **18** (*h*LDH5 IC_50_ = 245.7 µM, Figure S11A) interacted with the closed form of the *h*LDH receptor occupying the NADH binding site, whereas compound **19** (*h*LDH5 IC_50_ = 268.6 µM, Figure S11B) interacted with the open form of the receptor occupying the pyruvate binding site. As shown in Figure S11A, the N-phenylacetamide portion of compound **18** shows two H-bonds with G29 and D52 and lipophilic interactions with V26, V51, V53, A96, I116, F119 and I120. The isopropyl group is exposed to the solvent and does not show important interactions, whereas the benzyl carbamate portion of **18** shows a lipophilic interaction with V31 and two H-bonds with the backbone of G29 and G97. Differently from **18**, compound **19** interacts in the SBP of *h*LDH5 (Figure S11B); the 3,5 dinitrobenzene substituent of the molecule shows three H-bonds with the side-chain of R169, the hydroxyl group and the nitrogen backbone of T248, as well as lipophilic interactions with V241. The amide portion does not show important interactions, whereas the methyl-2-phenylacetate group showed lipophilic interactions with L109 and P139 and one H-bond with the nitrogen backbone of Q100.

In 2016, Braca and co-workers screened against *h*LDH5 a series of phenylpropanoids and flavonoids isolated from the aerial parts of *Phlomis kurdica* [32]. After biochemical evaluation, luteolin 7-*O*-β-d-glucopyranoside (**20**, Figure S12) showed a promising activity (*h*LDH5 IC_50_ = 139.2 µM, *K*_i_ = 68 µM in competition with NADH and *K*_i_ = 137 µM in competition with pyruvate) and its interaction with the enzyme was subjected to molecular modeling evaluation. This compound was docked inside the open conformation of *h*LDH5 in absence of NADH and pyruvate and the complex was subjected to five nanoseconds of MD simulation. Figure S12 shows the hypothetical binding mode of this compound. The ligand partially fills the cavity occupied by the nicotinamide riboside portion of NADH and it is stabilized by a large number of H-bonds and lipophilic interactions with the protein. The 3,4-dihydroxyphenyl group establishes two H-bonds with the oxygen backbone of S137 and the hydroxyl group of S161. The 5-hydroxy-4*H*-chromen-4-one central scaffold forms two H-bonds with the nitrogen backbone of T248 and the hydroxyl group of S249, whereas the glucoside portion of the ligand forms four additional H-bonds with the backbone of T95, A96, A98 and N138.

**Figure 9:**
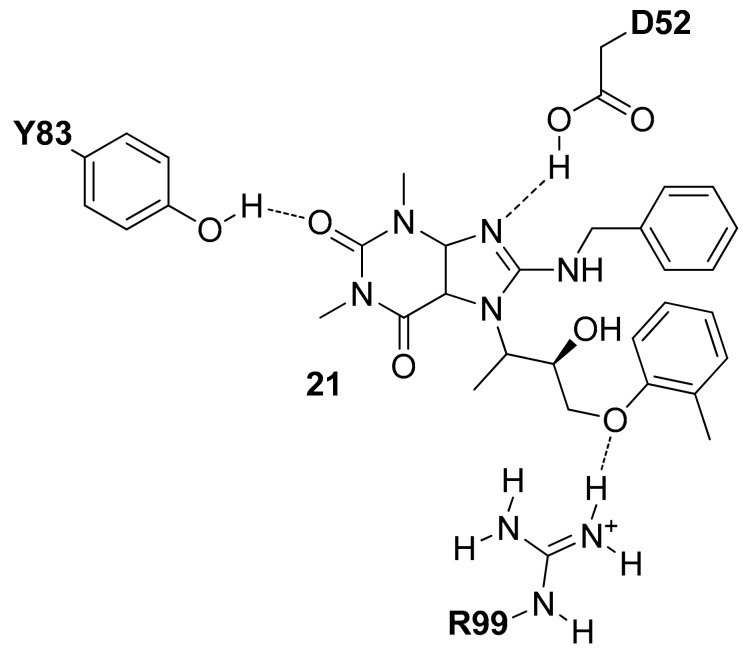
Schematic 2D representation of the **21**–*h*LDH5 H-bond interactions.

In 2016 Chen and co-workers applied a docking-based approach on 5688 compounds obtained filtering the ChemBridge commercial database [33]. The docked compounds were ranked based on their binding score and the presence of interactions with D52 (reported in the protonated form) and R99. Following these rules, four compounds were selected, purchased and tested for their *h*LDH5 inhibition properties and among them, the tetrahydro-1*H*-purine-2,6-dione derivative **21** (Figure 9) showed the most interesting activity (*h*LDH5 IC_50_ = 0.25 µM), confirmed also by antiproliferative cell tests. The authors hypothesized that this compound interacts with the open conformation of *h*LDH5 in the absence of NADH and pyruvate with the tetrahydro-1*H*-purine-2,6-dione central scaffold that shows H-bonds with the side-chain of D52 and Y83, whereas the *o*-tolyloxy substituent forms an H-bond with the side-chain of R99 (see Figure 9).

**Figure 10:**
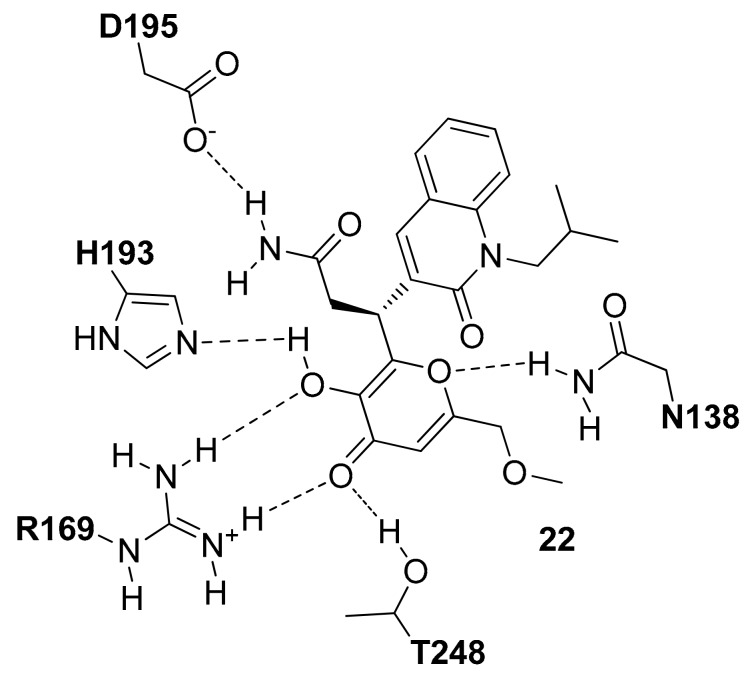
Schematic 2D representation of the **22**-*h*LDH5 H-bond interactions.

Recently, Fang and co-workers, starting from a pre-filtered commercial database of 8415 compounds, applied a docking-based VS study on the open conformation of *h*LDH5 in the presence of the NADH cofactor [34]. The compounds showing a total binding score higher than that of the reference co-crystallized inhibitor (PDB entry 4QO8 [20]) were further filtered selecting only those compounds that formed no less than two H-bonds with residues of N138, R169 and H193. Following these rules, seven compounds were purchased and tested for their LDH5 inhibition properties and among them, compound **22** (Figure 10) was the most promising as it showed an IC_50_ value of 2.37 µM and a *K*_d_ value of 0.95 µM. As shown in Figure 10, the center of the main interactions of compound **22** is the 3-hydroxy-4*H*-pyranone ring that forms H-bonds with the side-chain of N138, H193, D195 and T248. The methoxymethyl and the quinolinone fragments do not appear to show important interactions with the *h*LDH5 protein.

**Figure 11:**
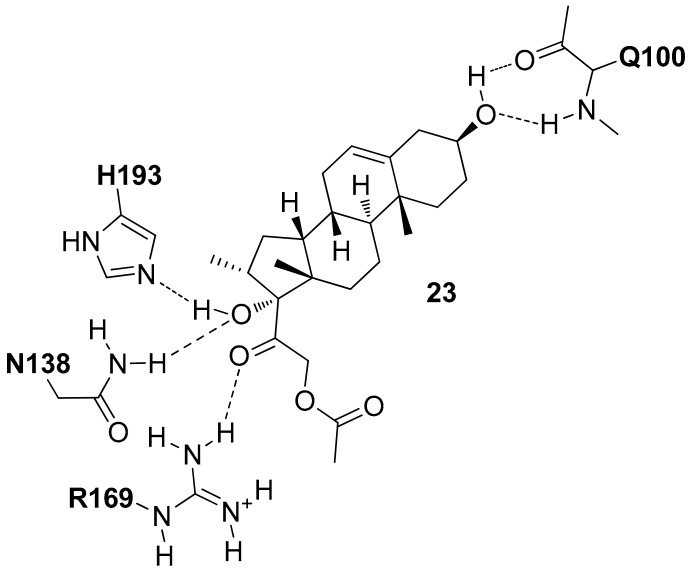
Schematic 2D representation of the **23**-*h*LDH5 H-bond interactions.

In 2017, Xiao and co-workers by using the open conformation of *h*LDH5 in the presence of NADH (PDB entry 4QO8 [20]), carried out a docking-based VS study [35]. A library with 16,000 compounds of diverse chemical structure downloaded from ZINC database was filtered in order to discard compounds with unfavorable physicochemical properties that did not meet the drug-like rules; then the remaining compounds were docked into the binding pocket by using the Surflex-Dock software. The compounds able to form H-bonds with the N138, R169 and H193 residues of *h*LDH5 were selected and following this procedure, six compounds were purchased and tested. As a result, all the six compounds showed inhibitory potency against *h*LDH5 and in particular compound **23** (Figure 11) showed the best activity (*h*LDH5 IC_50_ = 0.36 µM). The phenanthrenic large portion of this compound is important to allow the interaction of the two hydroxyl groups at the extremities of the central scaffold with the Q100 backbone and N138 and H193 side-chains. The acetate portion seems to be not important for the ligand–protein interaction, whereas the ketonic carbonyl oxygen forms an H-bond with R169 (Figure 11).

## 4. Conclusions

During the last eight years, great efforts from companies and academics have been made for identifying new *h*LDH5 inhibitors. The great interest associated with the inhibition of this enzyme can be ascribed to the novelty of this target, since up to 2010 [36] inhibition of *h*LDH5 was only considered as a side effect of compounds mainly developed as anti-malaria agents. In fact, the development of compounds selectively targeting the human isoform took quite a long time before it was able to be considered as a valid anticancer strategy. Moreover, the inhibition of this enzyme presumably causes only mild consequences to human health, since hereditary deficiency of the LDH5 subunit provokes myoglobinuria only after intense anaerobic exercise, whereas it does not generate any symptoms under ordinary circumstances [37].

The design and development of new *h*LDH5 inhibitors require the overcoming of different obstacles: (a) from a structural point of view, the *h*LDH5 binding site shows a certain level of flexibility and the presence of two possible conformations—open or closed—of the active-site loop; (b) the binding process of the substrate and the cofactor, though primarily driven by ionic interactions, shows affinities for the enzyme in the high micromolar order, thus making the identification of nanomolar inhibitors remarkably difficult; (c) the presence of different positive charges in the binding site has so far led to the development of negatively charged compounds, whose drawback is the poor cellular permeability, thus resulting in insufficient activities in cellular assays. However, despite these difficulties, by exploring the different sub-pockets—SBP, NBP and ABP—of the *h*LDH5 binding site some chemical classes of potent inhibitors have been successfully developed, displaying also cellular activities. The possibility of developing active *h*LDH5 inhibitors has been supported by the recent study of Malonely and co-workers, in which they reported the discovery and optimization of a novel series of pyrazole-based inhibitors. The lead compound of this series exhibited low nanomolar inhibition of *h*LDH5, submicromolar inhibition of lactate production, and inhibition of glycolysis in MIA PaCa2 pancreatic cancer and A673 sarcoma cells. The X-ray structures of this compound complexed with *h*LDH5 will be soon available and will add another important contribution for the development of *h*LDH5 inhibitors as prospective anticancer agents [38].

## Supplementary Materials

Supplementary Materials are available online.

## Abbreviations

The following abbreviations are used in this manuscript:

*h*LDH5	human lactate dehydrogenase
SBP	substrate binding pocket
NBP	nicotinamide binding pocket
ABP	adenine binding pocket
SPR	surface plasmon resonance
STD-NMR	saturation transfer difference NMR
VS	virtual screening
MD	molecular dynamic
