# Supplementary material for: Three-Dimensional Analysis of the Interactions between hLDH5 and Its Inhibitors
Source: Molecules. 2017 Dec 13;22(12):2217. doi: 10.3390/molecules22122217 (PMC6149858; doi:10.3390/molecules22122217)
Supplement: Supplementary file 1 [file molecules-22-02217-s001.pdf]

Supplementary Materials

Three-dimensional analysis of the interactions between *h*LDH5 and its inhibitors

Giulio Poli, Carlotta Granchi, Mohamed Aissaoui, Filippo Minutolo, Tiziano Tuccinardi
E-mail: tiziano.tuccinardi@unipi.it

Table of Contents

Figure S1. Superimposed structures of *h*LDH5 showing the large and small domains S2

Figure S2. Chemical structure of oxamate (**1**) and H-bond interactions S3

Figure S3. Chemical structure of **3** and X-ray structure of *h*LDH5 in complex with compound **3** S4

Figure S4. Chemical structure of **7** and X-ray structure of *h*LDH5 in complex with compound **7** S5

Figure S5. Chemical structure of **8** and X-ray structure of *h*LDH5 in complex with compound **8** S5

Figure S6. Chemical structure of **9** and X-ray structure of *h*LDH5 in complex with compound **9** S6

Figure S7. Chemical structure of **10** and X-ray structure of *h*LDH5 in complex with compound **10** S6

Figure S8. Chemical structure of **11** and X-ray structure of *h*LDH5 in complex with compound **11** S7

Figure S9. Chemical structure of **12** and X-ray structures of *h*LDH5 in complex with compound **12** S8

Figure S10. Chemical structure of **14**, **15** and X-ray structures of *h*LDH5 in complex with **14** and **15** S9

Figure S11. Schematic 2D representation of the **18**-*h*LDH5 and **19**-*h*LDH5 H-bond interactions S10

Figure S12. Schematic 2D representation of the **20**-*h*LDH5 H-bond interactions S10

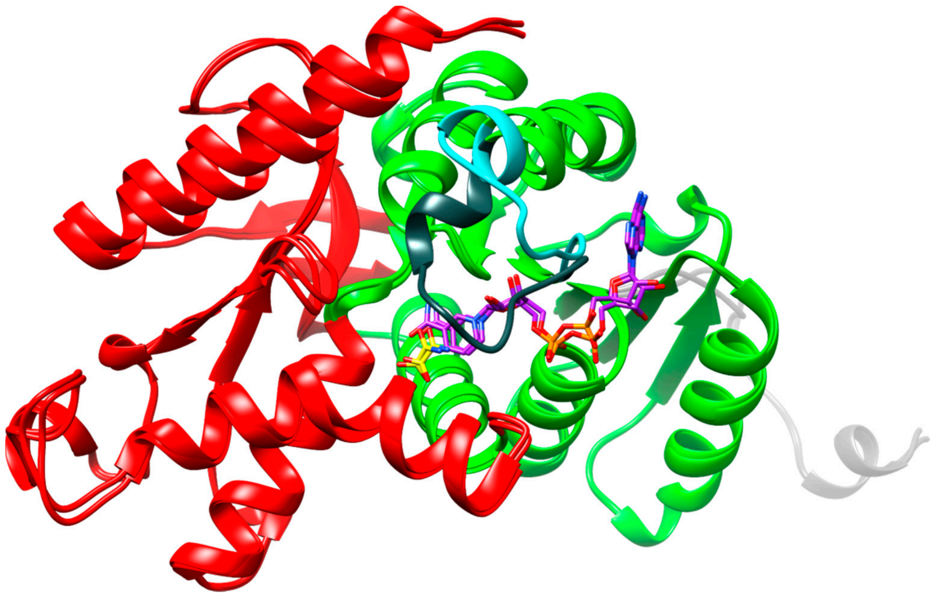

Figure S1. Superimposed structures of *h*LDH5 showing the large (green) and small (red) domains (PDB code 1I10). The active-site loop in the open (sky-blue) and closed (dark slate gray) conformation is highlighted. NADH and oxamate (**1**) are shown in purple and yellow, respectively.

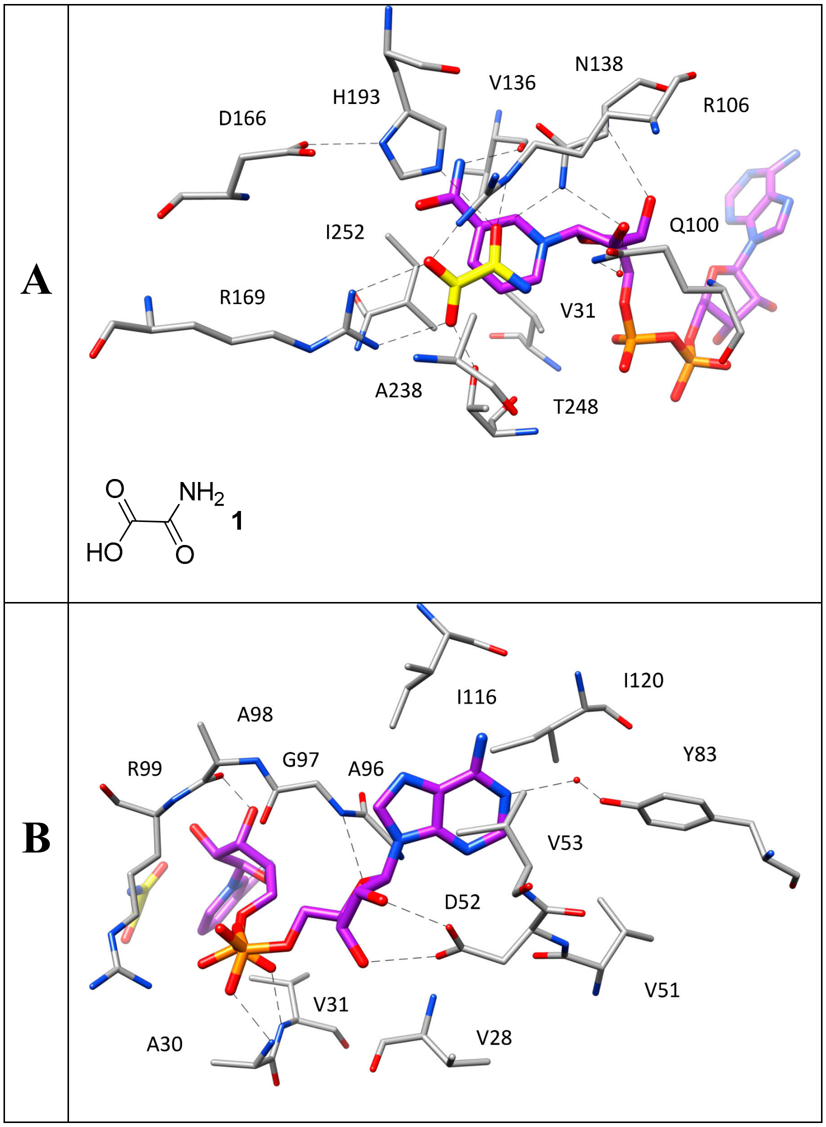

Figure S2. Chemical structure of oxamate (**1**) and H-bond interactions with *h*LDH-A identified in close proximity to the nicotinammide fragment of NADH, **1** and the residues delimiting the SBP and NBP (A), or between NADH and the residues delimiting the ABP (B) (PDB code 1I10). NADH and **1** are shown in purple and yellow, respectively.

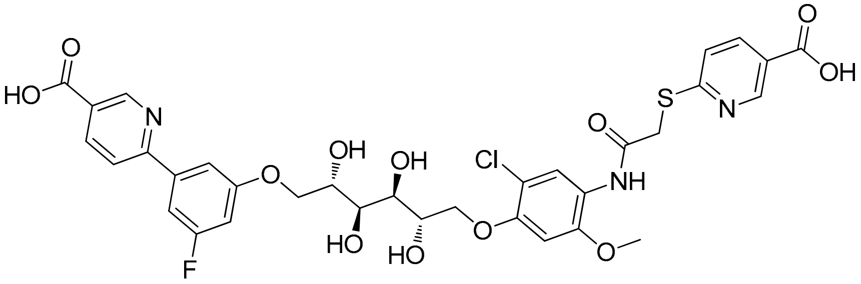

3

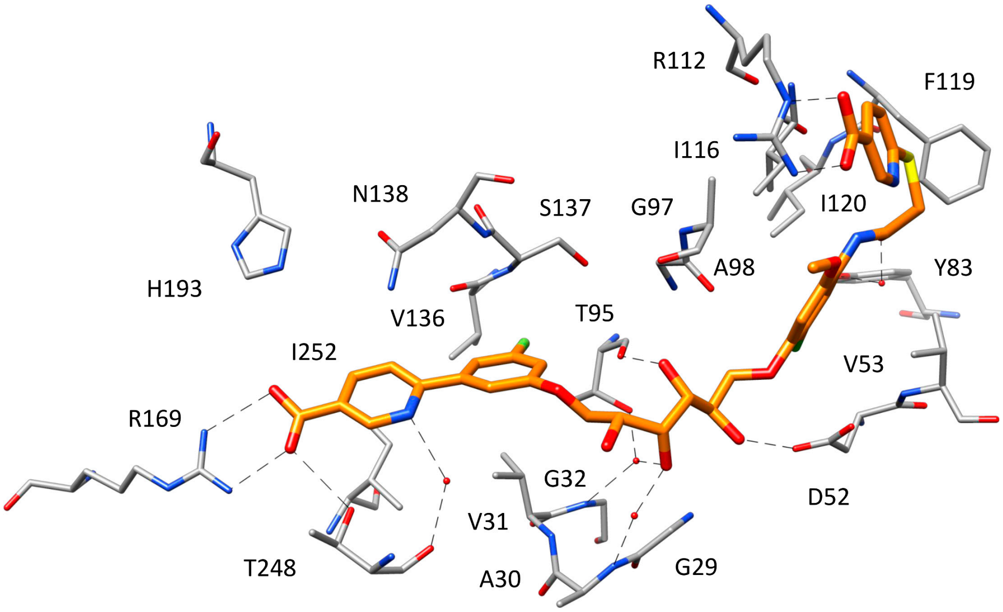

Figure S3. Chemical structure of **3** and X-ray structure of *h*LDH5 in complex with compound **3** (PDB code 4I9H). The active site residues interacting with the inhibitor are shown and the ligand-protein H-bonds are highlighted.

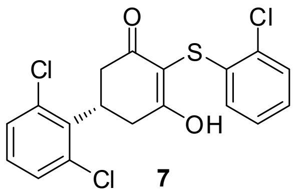

7

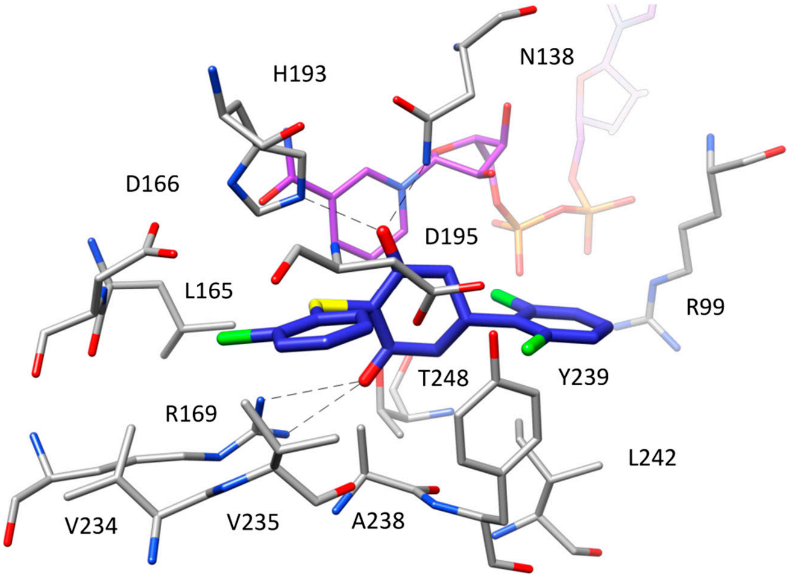

Figure S4. Chemical structure of **7** and X-ray structure of hLDH5 in complex with compound **7** (PDB code 4QO8). The active site residues interacting with the inhibitor are shown and the ligand-protein H-bonds are highlighted.

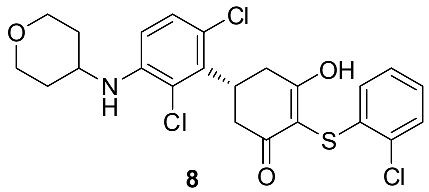

8

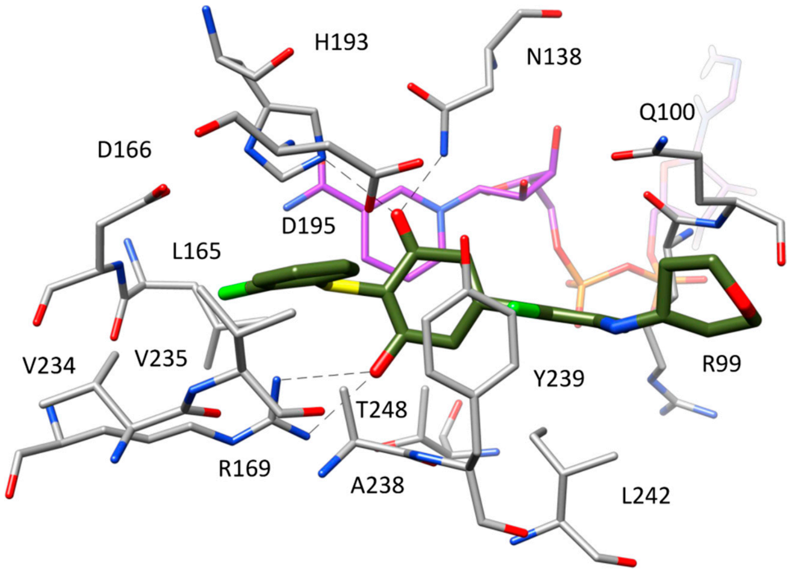

Figure S5. Chemical structure of **8** and X-ray structure of hLDH5 in complex with compound **8** (PDB code 4R69). The active site residues interacting with the inhibitor are shown and the ligand-protein H-bonds are highlighted.

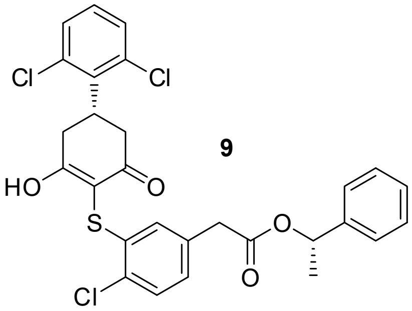

9

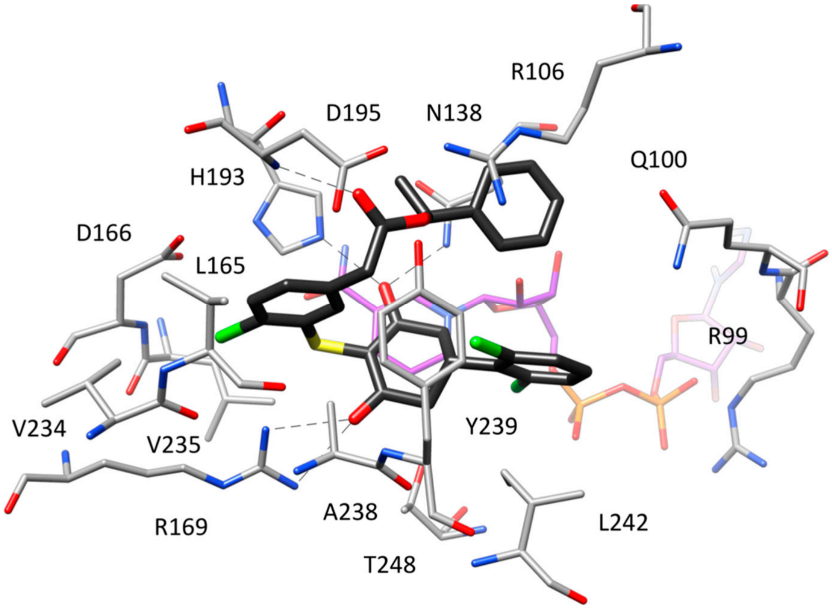

Figure S6. Chemical structure of **9** and X-ray structure of hLDH5 in complex with compound **9** (PDB code 4R68). The active site residues interacting with the inhibitor are shown and the ligand-protein H-bonds are highlighted

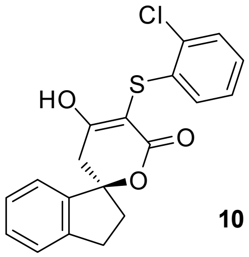

10

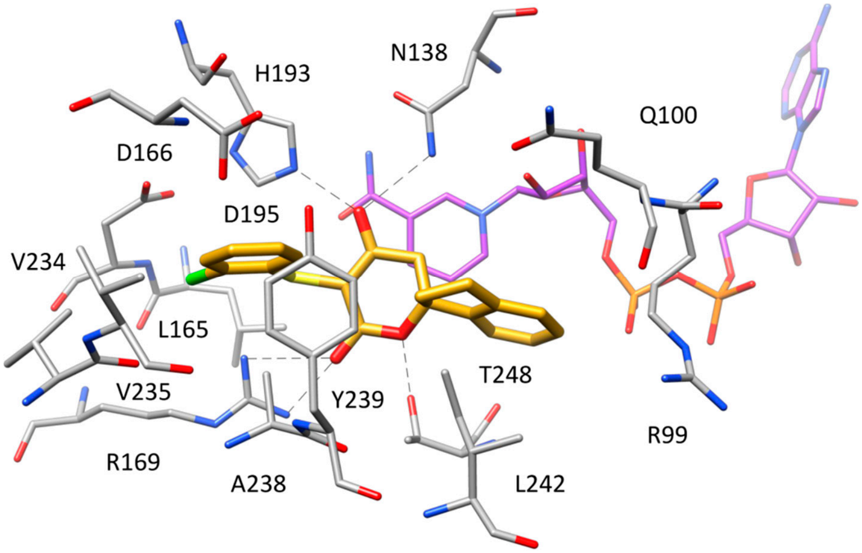

Figure S7. Chemical structure of **10** and X-ray structure of hLDH5 in complex with compound **10** (PDB code 4RLS). The active site residues interacting with the inhibitor are shown and the ligand-protein H-bonds are highlighted.

11

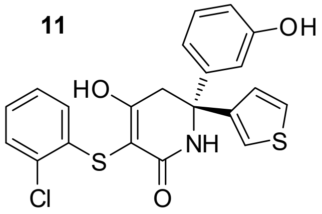

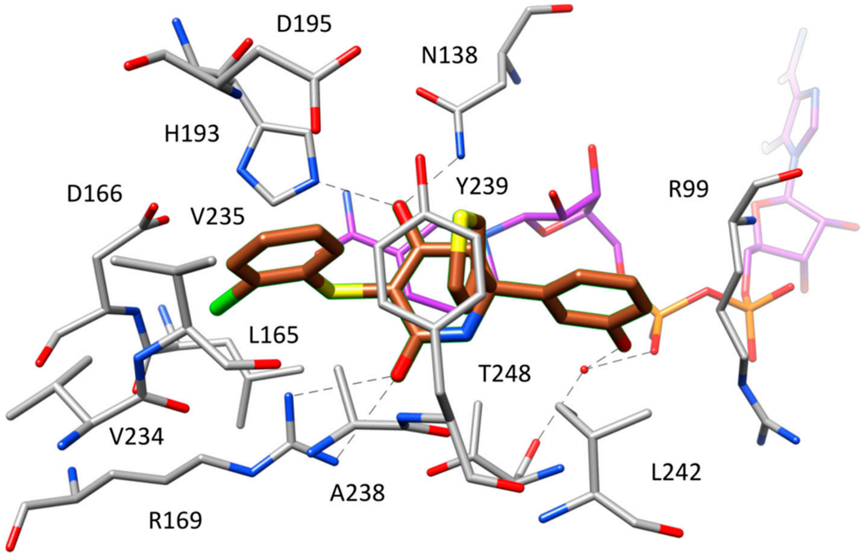

Figure S8. Chemical structure of **11** and X-ray structure of hLDH5 in complex with compound **11** (PDB code 5IXS). The active site residues interacting with the inhibitor are shown and the ligand-protein H-bonds are highlighted

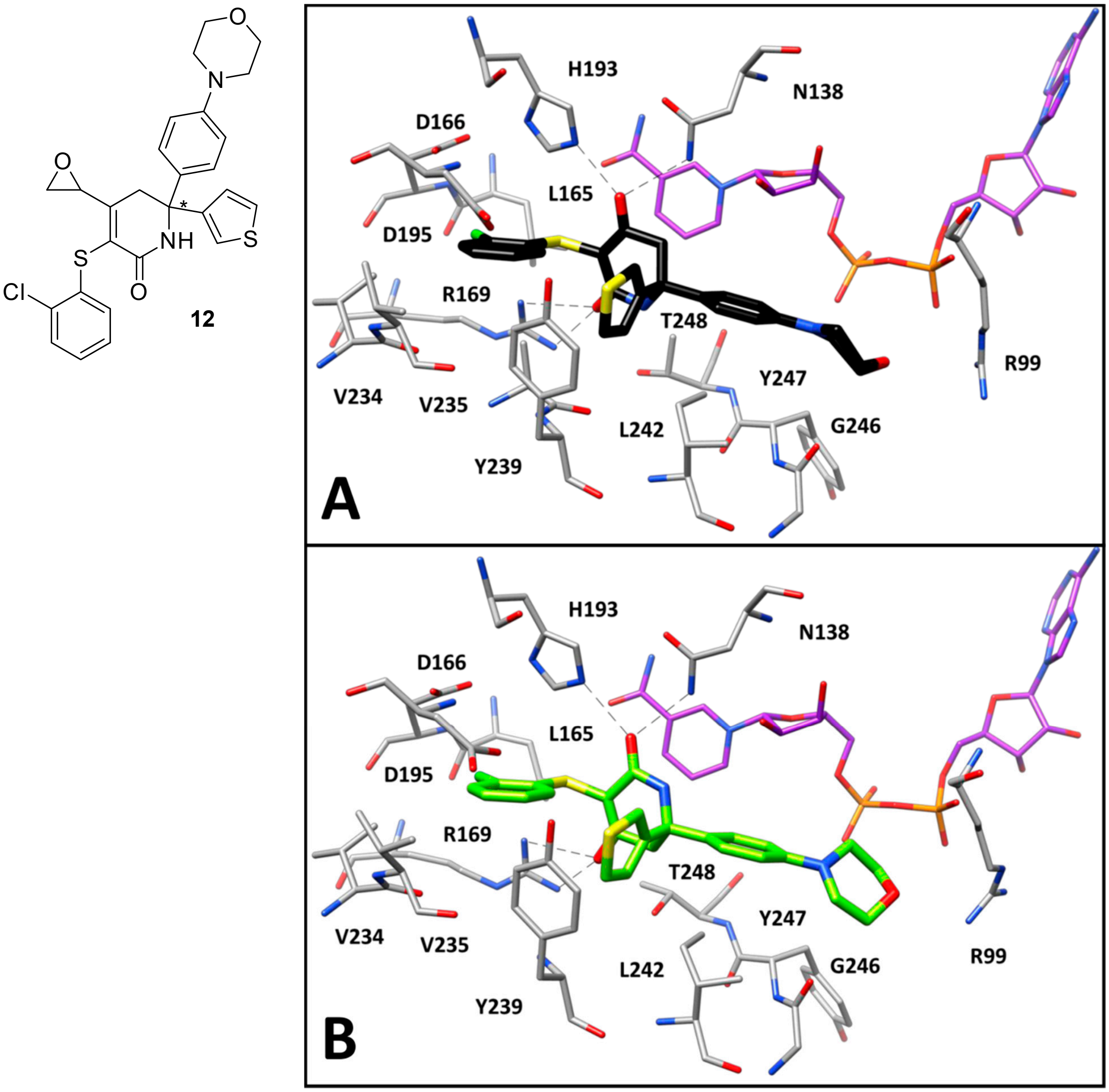

Figure S9. Chemical structure of **12** and (A) X-ray structures of *h*LDH5 in complex with compound *R*-**12** (PDB code 4ZVV); (B) X-ray structures of *h*LDH5 in complex with compound *S*-**12** (PDB code 5IXY). The active site residues interacting with the inhibitors are shown and the ligand-protein H-bonds are highlighted.

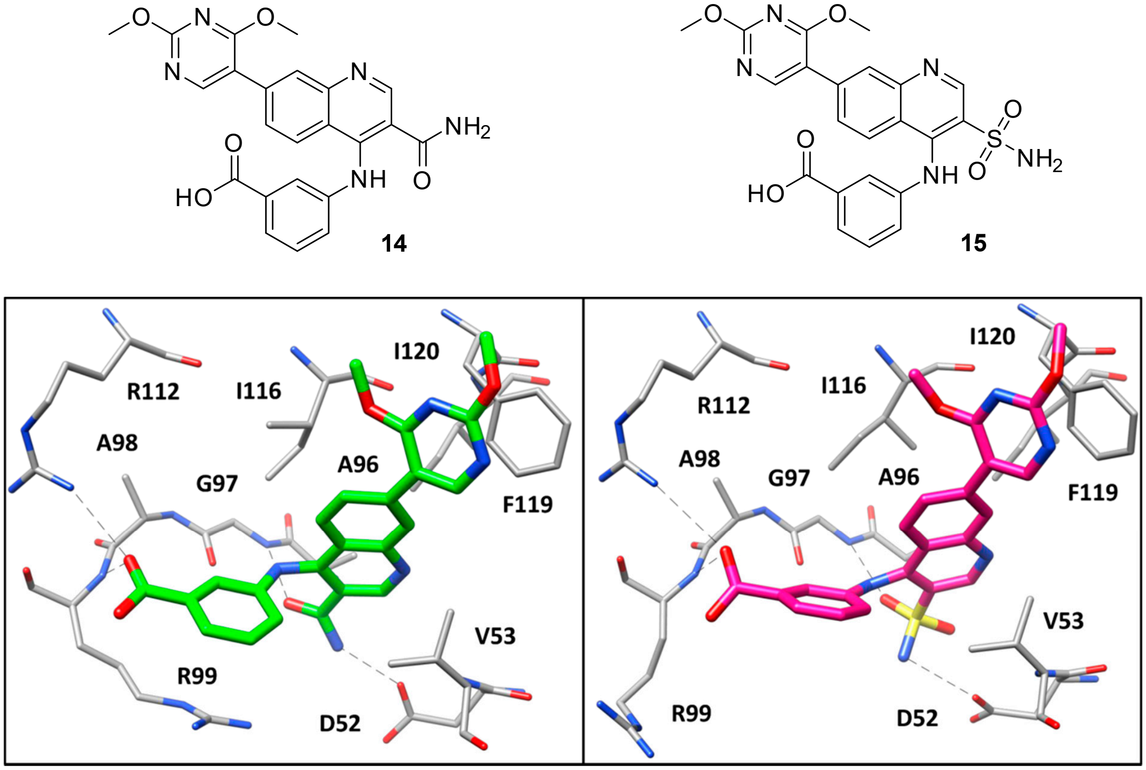

Figure S10. Chemical structure of **14** and **15** and (A) X-ray structures of *h*LDH5 in complex with compound **14** (PDB code 4QT0) and (B) compound **15** (PDB code 4QSM). The active site residues interacting with the inhibitors are shown and the ligand-protein H-bonds are highlighted.

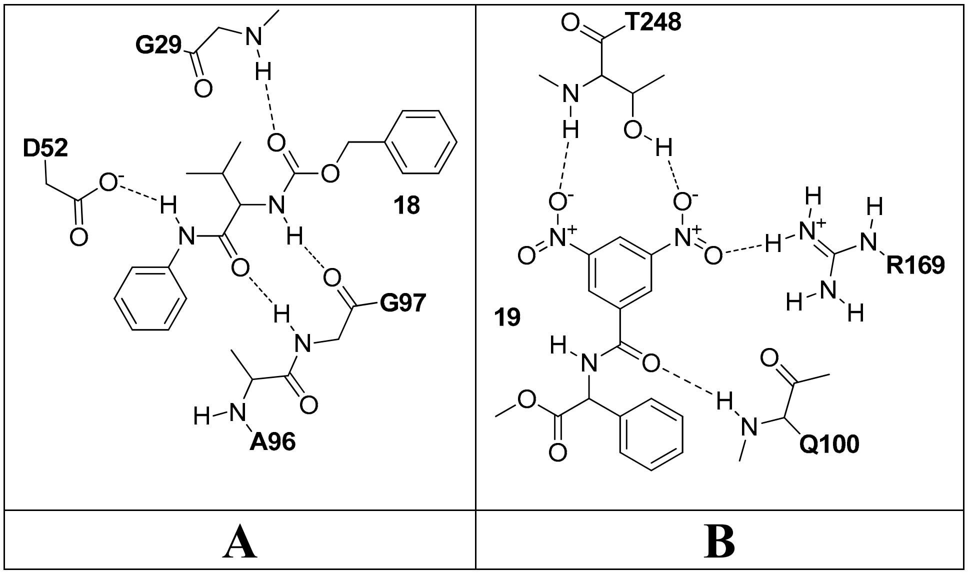

Figure S11. Schematic 2D representation of the **18**-LDH5 (1) and **19**-LDH5 (2) H-bond interactions.

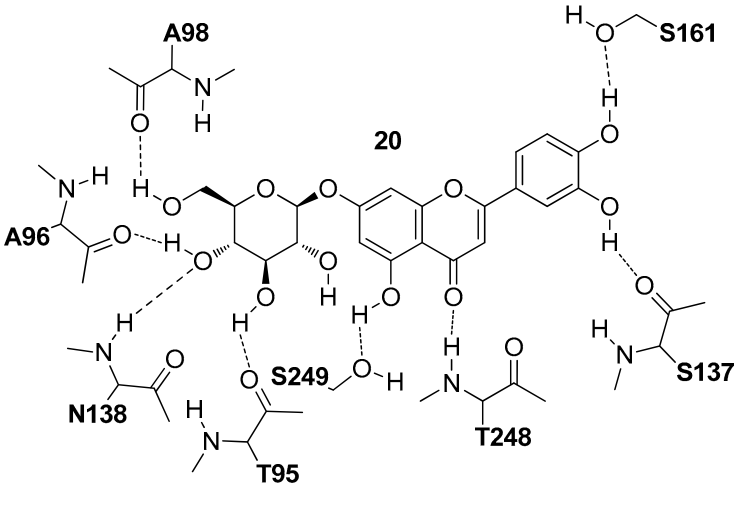

Figure S12. Schematic 2D representation of the **20**-LDH5 H-bond interactions.
